# p73 regulates maintenance of neural stem cell

**DOI:** 10.1016/j.bbrc.2010.10.087

**Published:** 2010-12-03

**Authors:** Massimiliano Agostini, Paola Tucci, Hailan Chen, Richard A. Knight, Daniele Bano, Pierluigi Nicotera, Frank McKeon, Gerry Melino

**Affiliations:** a Medical Research Council, Toxicology Unit, Leicester University, Leicester LE1 9HN, UK; b Biochemistry Laboratory, IDI-IRCCS, C/O University of Rome “Tor Vergata”, 00133 Rome, Italy; c Deutsche Zentrum für Neurodegenerative Erkrankungen (DZNE), Bonn, Germany; d Department of Cell Biology, Harvard Medical School, Boston, MA 02115, USA

**Keywords:** TAp73, transcriptionally active p73, ΔNp73, amino truncated p73, −/−, knockout mice, DIV, day in vitro, DMED, Dulbecco minimal essential medium, FBS, foetal bovine serum, EGF, epidermal growth factor, *C*_t_, threshold cycle, p73, Neural stem cell, Neurogenesis, Self-renewal, p53 family, Apoptosis

## Abstract

p73, a member of the p53 family, is a transcription factor that plays a key role in many biological processes. In the present study, we show that TAp73 is expressed in neural stem cells (NSC) and its expression increases following their differentiation. NSC from p73 null mice have a reduced proliferative potential, together with reduced expression of members of the Sox-2 and Notch gene families known to be important for NSC proliferation. In parallel with this in vitro data, the width of the neurogenic areas was reduced in the brains of embryonic and adult p73−/− mice. These data suggest that p73, and in particular TAp73, is important for maintenance of the NSC pool.

## Research highlights

► TAp73 is expressed in neural stem cells and its expression increases following their differentiation. ► Neural stem cells from p73 null mice have a reduced proliferative potential. ► p73-deficient neural stem cells show reduced expression of members of the Sox-2 and Notch gene families. ► Neurogenic areas are reduced in the brains of embryonic and adult p73−/− mice.

## 1 Introduction

Embryonic and adult neural stem cells (NSC) are precursors that retain the capacity to proliferate and produce identical daughter cells (self-renewal), and also the ability to differentiate into neurons, astrocytes and oligodendrocytes [1]. The process of self-renewal plays a key role in the development, as well as in the preservation of adult tissues. The regulation of this process requires that the multiple pathways involved in the regulation of proliferation and/or maintenance of the undifferentiated phenotype are tightly coordinated. Pathways that control differentiation and apoptosis are also involved in self-renewal [2].

The p53 family which includes p53 itself, p63 and p73 are transcription factors that play key roles as regulators of proliferation, differentiation, cell death, stem cell renewal and cell fate commitment [3–5]. So far, p53 and ΔNp63 are the only members of the family that have been reported to play a role in NSC [6,7].

Like other members of the p53 family, the Trp73 gene encodes a protein composed of a DNA-binding domain, a transactivation (TA) domain and an oligomerization domain. However, because of the presence of two distinct promoters, Trp73 is expressed as two major isoforms either containing (TAp73) or not (ΔNp73) the TA domain. As a result, TAp73 and ΔNp73 have distinct functions, being, for example, proapoptotic [8,9] or antiapoptotic [10], respectively. Furthermore, it has been shown that p73 can induce neurite outgrowth and expression of neuronal markers in neuroblastoma cell lines [11] and in primary oligodendrocytes [12] indicating a possible role in brain development. Indeed, p73-deficient mice manifest complex defects in neuronal development [13], such as congenital hydrocephalus, hippocampal dysgenesis with the lower blade of dentate gyrus truncated or missing, together with defects in pheromone detection. This phenotype is conserved in the selective TAp73 [14] and ΔNp73 [15] knockouts mice.

In the present study, we have investigated the possibility that p73 could play a role in NSC biology. Using the well-established in vitro system of embryonic NSC [16] we identify p73 as a positive regulator of these cells since neurospheres derived from p73−/− mice proliferate less than wild-type neurospheres and show dysregulation of several genes involved in NSC self-renewal.

## 2 Materials and methods

### 2.1 Mice

The p73−/− mice were generated as previously described [13]. Mice were bred and subjected to listed procedures under the Project Licence released from the Home Office.

### 2.2 Neurosphere culture

Neural stem cells were obtained from mice at gestational day E14. Briefly, cortex was dissected and triturated until a single cell suspension was achieved. Cells were counted and plated at 2 × 10^6^ cells/10 ml/25 cm^2^ flask in NeuroCult® NSC Basal Medium (Mouse) with proliferation supplements and rh-EGF (10 ng/ml) (all purchased from StemCells Technologies) following the manufacturer’s instructions, and this was considered passage 0. Neurospheres were allowed to form for 7 days. Neurospheres were passaged every 5 days. Passage 1 neurospheres were dissociated with NeuroCult® chemical dissociation kit (StemCells Technologies) and replated in triplicates at clonal density (20 × 10^3^ cells/ml in 24-well plate) and regeneration of new neurospheres was monitored. At the times indicated, the number and size of neurospheres and the total number of cells was analyzed. For the differentiation of NSC, a single cell suspension (5 × 10^5^ cells/well/24-well plate) was plated on the poly-l-ornithine glass coverslips in NeuroCult® NSC Basal Medium (Mouse) with differentiation supplements.

### 2.3 RNA extraction and real-time PCR

Total RNA from cells was isolated using Trizol (Invitrogen) according to the manufacturer’s instructions. RNA samples were treated with RNase-free DNase I (Sigma). Total RNA was reverse transcribed using Superscript III reverse transcriptase and oligo(dT) primer (Invitrogen). qRT-PCR was performed in ABI PRISM 7000 Sequence Detection System (Applied Biosystem) with SYBR green ready mix (Applied Biosystem) and specific primers: TAp73 Fwd 5′-GCACCTACTTTGACCTCCCC-3′ and Rev 5′-GCACTGCTGAGCAAATTGAAC-3′; ΔNp73 Fwd 5′-ATGCTTTACGTCGGTGACCC-3′, and Rev 5′-GCACTGCTGAGCAAATTGAAC-3′; p53 Fwd 5′-CTCTCCCCCGCAAAAGAAAAA-3′ and Rev 5′-CGGAACATCTCGAAGCGTTTA-3′; GAPDH Fwd 5′-CAATGAATACGGCTACAGCAAC-3′ and Rev 5′-AGGGAGATGCTCAGTGTTGG-3′; Sox-2 For 5′-TCCAAAAACTAATCACAACAATCG-3′ and Rev 5′-GAAGTGCAATTGGGATGAAAA-3′; Hes-1 For 5′-ACACCGGACAAACCAAAGAC-3′ and Rev 5′-CGCCTCTTCTCCATGATAGG-3′; Hes-5 For 5′- GATGCTCAGTCCCAAGGAGA-3′ and Rev 5′-AGCTTCAGCTGCTCTATGCTG-3′; Nanog For 5′-CACCCACCCATGCTAGTCTT-3′ and Rev 5′-ACCCTCAAACTCCTGGTCCT-3′; Notch-1 For 5′-ACTATCTCGGCGGCTTTTC-3′ and Rev 5′-GGCACTCGTTGATCTCCTCT-3′.

The expression of each gene was defined from the threshold cycle (*C*_t_), and relative expression levels were calculated by using the $2^{-\Delta \Delta C_{\text{t}}}$ method after normalization with reference to expression of the housekeeping gene GAPDH.

### 2.4 Immunofluorescence

E17.5 and P7 brains were fixed in 4% paraformaldehyde and embedded in paraffin. Coronal sections were de-waxed, re-hydrated in graded alcohols and rinsed in distilled water. The sections were blocked in 10% goat serum in 0.1% PBS–Tween (vol/vol), and then incubated with primary and secondary antibodies in blocking buffer. Cell nuclei were stained with Hoechst 33342 (Sigma) and mounted for confocal analysis. Two different images of each section were taken and the number of cells labeled was counted (*n* = 2 p73−/− compared to *n* = 2 control littermates). The following primary antibodies were used: nestin (mouse, 1/50, Millipore), GFAP (rabbit, 1/500, Dako).

### 2.5 Statistical analysis

All results are expressed as means ± SD. *P* *⩽* 0.05 was considered significant.

## 3 Results and discussion

### 3.1 TAp73 is expressed in NSC and is up-regulated during neurosphere differentiation

**Fig. 1:**
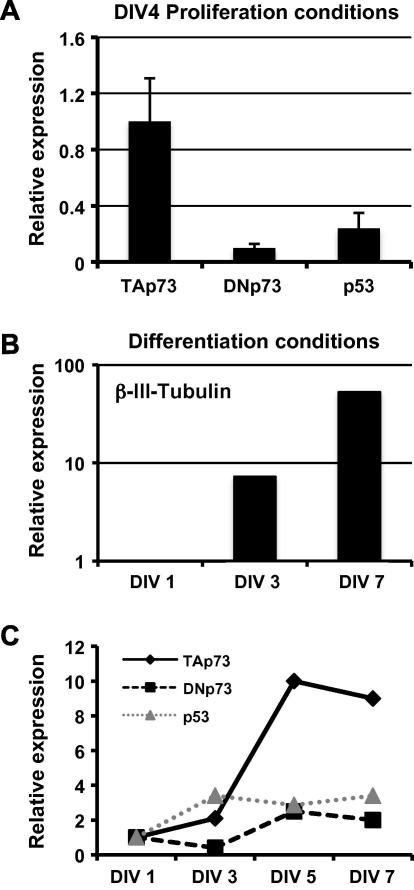
Expression of p53 family members in NSC. (A) NSC were cultured as described in Section 2. Cells were harvested at the indicated time points and expression of TAp73, ΔNp73 and p53 was evaluated by qPCR. Data are normalized to the housekeeping gene Gapdh and are relative to the expression of TAp73. Data represent mean ± SD of three different experiments. (B and C) An NSC single cell suspension was allowed to differentiate in vitro and expression of the indicated genes was evaluated by qPCR. Data are normalized to the housekeeping gene Gapdh relative to DIV 1. A representative experiment of three independent experiments is shown.

In order to investigate whether p73 could play a role in the biology of NSC, we first evaluated its expression under two different conditions. Wild-type (WT) NSC were isolated from E14.5 embryos and maintained in culture either under proliferating conditions, or induced to differentiate. After 4 days in vitro (DIV), cells were harvested and total RNA was isolated. qPCR analysis (Fig. 1A) shows that TAp73 is the most abundant member of the p53 family expressed under proliferative conditions, and TAp73 expression is roughly 10-fold greater than that of ΔNp73 and 3-fold higher than that of p53. Next, we assayed the expression of these three proteins during differentiation of neurospheres. After deprivation of mitogenic stimuli, WT neurospheres undergo differentiation as shown by the appearance of cells positive for the neuronal marker β-III-Tubulin (Fig. 1B). As shown in Fig. 1C, under these differentiating conditions, we observed a marked increase in expression of TAp73 with only minor changes in expression of ΔNp73 and p53. Together, these data suggest that TAp73 is mechanistically involved in the biology of NSC, and confirm previous observations [11] that TAp73 play a role in neuronal differentiation. In addition, we show for the first time that TAp73 is more prominently expressed than ΔNp73 in the neural lineage.

### 3.2 p73-deficient NSC show decreased proliferation

**Fig. 2:**
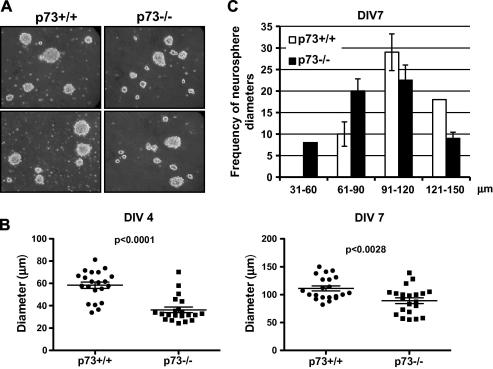
p73-deficient NSC show decreased proliferation. (A) E14.5 neurospheres were derived from wild-type and p73−/− mice. A representative (two different fields per each genotyping) picture of NSC is shown. (B) Diameter of NSC from p73+/+ and p73−/− mice at DIV 4 and 7, respectively. (C) Size distribution of NSC at DIV 7. p73−/− NSC accumulate in the lower range. Data represent mean ± SD of three different experiments.

Next, employing the neurospheres assay, we focused on whether p73 is required for proliferation and self-renew of embryonic NSC. After 7 days in culture, NSC from both WT and p73−/− mice formed neurospheres, but those derived from p73−/− mice were morphologically smaller (Fig. 2A) and the mean diameter of p73−/− neurospheres was significantly smaller than that of WT mice at both DIV 4 and DIV 7 (Fig. 2B). Moreover, as shown in Fig. 2C, p73−/− neurospheres tended to accumulate in the lower size range. These data suggest that p73 is required for optimum proliferation of neurospheres.

### 3.3 Loss of p73−/− impairs expression of genes involved in NSC self-renewal

**Fig. 3:**
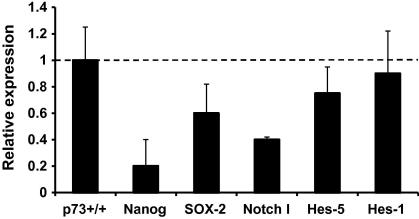
Loss of p73 impairs expression of genes involved in NSC self-renewal. Relative expression of the indicated genes in neurospheres derived from p73−/− mice at DIV 7. Data are normalized to the housekeeping gene Gapdh and relative to their expression in p73+/+ NSC. Data represent mean ± SD of three different experiments.

We next investigated the molecular mechanisms underlying the altered phenotype of p73−/− derived neurospheres. Pathways involving genes of the Sox [17] and Notch [18] families have been implicated in the proliferation/self-renewal of NSC. We therefore isolated RNA from DIV 7 neurospheres derived from WT and p73−/− mice and analyzed expression of candidate genes of these families by qPCR. As shown in Fig. 3 the absence of p73 results in a significant reduction in Sox-2, Notch-1 and Nanog expression, indicating that p73 directly or indirectly regulates these factors. Indeed, Sox-2−/− mice have a phenotype similar to that of p73−/− mice with a moderate lateral ventricle enlargement and a reduction of the posterior ventrolateral cortex. In addition, in the hippocampus the dentate gyrus is essentially absent [19].

### 3.4 Neurogenic zones are reduced in p73−/− mice

**Fig. 4:**
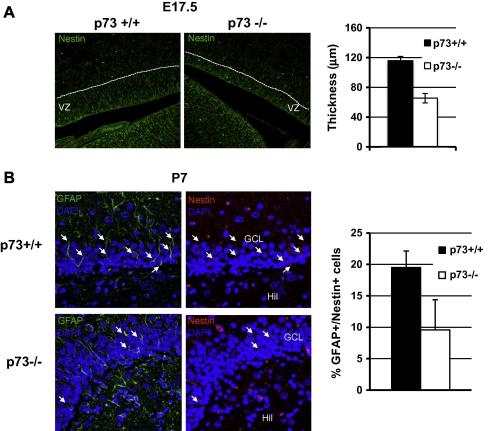
Reduction of neurogenic areas in p73−/− mice. (A) Nestin immunofluorescence of the ventricular zone in coronal sections in E17.5 mice. Magnification 20×. Graph shows the mean ± SD of thickness of neurogenic area. (B) The dentate gyrus from P7 p73+/+ and p73−/− mice was stained with antibodies to GFAP and nestin. Arrows indicate double positive cells. Magnification 40×. Graph shows the mean ± SD of GFAP/nestin positive cells. VZ = ventricular zone; GCL = granular cell layer; Hil = hilus.

The ventricular/subventricular zone and the dentate gyrus in the hippocampus are the neurogenic areas in embryonic and in adult mice respectively [20,21]. To investigate whether these zones were morphologically smaller in vivo, in parallel with the reduced proliferation of p73−/− derived neurospheres in vitro, we stained E17.5 brains from WT and p73−/− mice with anti-nestin antibodies. The results in Fig. 4A show that the width of the ventricular zone, the neurogenic area, is reduced in embryonic p73−/− mice when compared to WT mice. Similarly, the numbers of GFAP and nestin double positive cells in the dentate gyrus, the source of neurogenesis in the adult, are reduced in P7 p73−/− mice (Fig. 4B and C).

## 4 Conclusion

Two very recent reports [22,23] are in line with our results, providing an independent confirmation of the involvement of p73 in neuronal stemness.

Together, these data support an important role for p73, and particularly TAp73, in the proliferative potential of NSC and in their differentiation, probably by modulating expression of components of the Sox-2 and Notch pathways known to be involved in NSC proliferation. This requirement for p73 is apparent, both from the reduced size of neurospheres derived from p73−/− mice in vitro, and in the reduced thickness of brain areas responsible for neurogenesis in both embryonic and adult knockout mice in vivo. However, whether all the morphological and functional deficits in the brains of p73−/− mice arise from these actions of p73 on NSC or whether p73 exerts other independent effects has yet to be established.

## Conflict of interest

The authors declare no competing financial interests.
